# Supplementary material for: The genome sequence of the Marbled Brown, Drymonia dodonaea (Denis & Schiffermüller), 1775 (Lepidoptera: Notodontidae)
Source: Wellcome Open Res. 2025 Sep 9;10:498. [Version 1] doi: 10.12688/wellcomeopenres.24797.1 (PMC12531619; doi:10.12688/wellcomeopenres.24797.1)
Supplement: Peer review history

# Reviewer report: Reviewer response for version 1

**DOI:** 10.21956/wellcomeopenres.27320.r132871

**Published:** 2025-10-16

**Authors:** Sarah Inwood

**Affiliations:** 1 University of Otago, Dunedin, New Zealand

The authors have generated a high quality genome assembly for the Marbled Brown moth *Drymonia dodonaea*. This involved using state of the art PacBio HiFi sequencing alongside Hi-C sequencing to generate a chromosome-level scaffolded assembly. Quality control metrics for this assembly meet literature standards, with an N50 of 12 Mb, 99.89% of the assembled sequence being assigned to chromosomal-level scaffolds, above 95% k-mer completeness, and a high 98.7% of lepidopteran BUSCO orthologs being found within the assembly. Alongside this a mitochondrial genome was also assembled, with a length of 16.37 kb.

 Minor comments:

 -Length of the assembled mictochondrial genome could be stated in the results of the paper where it is reported that the mitochondrial genome was also assembled

 -Hifiasm, purge_dups, YaHS, and MitoHiFi missing from software table, therefore version number used is not stated

 -Conversely the table includes BEDTools, Cooler, fasta_windows and GoaT CLI which are never mentioned in text - were these tools used for this assembly and if so, what were they used for?

Are sufficient details of methods and materials provided to allow replication by others?

Partly

Is the rationale for creating the dataset(s) clearly described?

Yes

Are the datasets clearly presented in a useable and accessible format?

Yes

Are the protocols appropriate and is the work technically sound?

Yes

Reviewer Expertise:

Genomics, host-parasite interactions, parasitoid wasps, insects, viruses, biocontrol

I confirm that I have read this submission and believe that I have an appropriate level of expertise to confirm that it is of an acceptable scientific standard.

# Reviewer report: Reviewer response for version 1

**DOI:** 10.21956/wellcomeopenres.27320.r132873

**Published:** 2025-10-16

**Authors:** Kashif Zahoor

**Affiliations:** 1 Government College University Faisalabad, Faisalabad, Punjab, Pakistan

The article titled “The genome sequence of the Marbled Brown, *Drymonia dodonaea *(Denis & Schiffermüller), 1775 (Lepidoptera: Notodontidae)” describes genome of Marbled Brown Moth, *Drymonia dodonaea*. Its appreciable that mitochondrial genome has also been assembled. Its very well performed scientific work, well written, very well explained and beautifully presented with Figures and Tables. The work shows huge work rather I should say hard work. Authors covered all the aspects that there’s no such room to criticize or even to make critical comments. The article fully merits to be indexed, and I strongly recommend to get this indexed in your good journal.

 There’s only 2-3 points I want to raise:

  

- Is there any possibility to get more than these data; 25.37Gb from 2.61 million reads PacBio??? and 151.63 Gb from 1 004.20 million reads through Hi-C??? It seems likely that there could be more data if one may use any other technique/tool?
- Hi-C shows more reads but less data???
- PacBio has less reads but more gigabases???
- Is there any kind of optimum value for QV?

Are sufficient details of methods and materials provided to allow replication by others?

Yes

Is the rationale for creating the dataset(s) clearly described?

Yes

Are the datasets clearly presented in a useable and accessible format?

Yes

Are the protocols appropriate and is the work technically sound?

Yes

Reviewer Expertise:

Genetics, Molecular Biology, Entomology, Zoology

I confirm that I have read this submission and believe that I have an appropriate level of expertise to confirm that it is of an acceptable scientific standard.

# Reviewer report: Reviewer response for version 1

**DOI:** 10.21956/wellcomeopenres.27320.r132874

**Published:** 2025-09-27

**Authors:** Alexandra Cerqueira De Araujo

**Affiliations:** 1 University College London, London, UK

The authors present here a chromosome-level *de novo* assembly of the genome of *Drymonia dodonaea *(Notodontidae, Lepidoptera). This is the first assembly available for this species. This assembly is composed of 31 chromosome-level pseudomolecules (including a sex chromosome Z) and one mitochondrial chromosome. The assembly has a total length of 330.51 Mb and a N50 of 12.0 Mb. This assembly achieves a EBP reference standard of 6.C.Q62 and contains most of the orthologous genes found in lepidopterans (i.e. 98.7% of the complete BUSCO sequences). The quality of this assembly meets the recommended reference standard for genome assemblies, with no identifiable major issues. All methods used to produce this data is reported in this manuscript and the code is available here https://github.com/sanger-tol.

 Minor comments:

 1- in "Genome assembly"

 “Contigs were further scaffolded with Hi-C data in YaHS” is a bit ambiguous, may be consider changing “in YaHS” to “using YaHS” as it could sound like the Hi-C data used was stored in YaHS.

 2- Regarding the number of chromosomal pseudomolecules, although other chromosome-level assemblies of species of the same genus have between 29 to 31 chromosomes, is there any karyotype data supporting it? (this question does not invalidate the results found by the authors, this is pure curiosity)

Are sufficient details of methods and materials provided to allow replication by others?

Yes

Is the rationale for creating the dataset(s) clearly described?

Yes

Are the datasets clearly presented in a useable and accessible format?

Yes

Are the protocols appropriate and is the work technically sound?

Yes

Reviewer Expertise:

Comparative genomics

I confirm that I have read this submission and believe that I have an appropriate level of expertise to confirm that it is of an acceptable scientific standard.
